# Pathogenic dengue virus TW2015 strain infection triggers anaerobic glycolysis and enhances mortality in diabetic mice

**DOI:** 10.1128/jvi.01177-25

**Published:** 2025-11-10

**Authors:** Yi-Ping Kuo, Shih-Syong Dai, En-Ju Lin, Wann-Neng Jane, Wei-Hsiang Tsai, Wan-Ting Tsai, Zaida Nur Imana, Wan-Ju Tung, Yu-Siang Su, Chih-Feng Tien, Chia-Yi Yu, Chun-Hong Chen, Guann-Yi Yu

**Affiliations:** 1 National Institute of Infectious Diseases and Vaccinology, National Health Research Institutes 50115, Miaoli, Taiwan; 2 Institute of Plant and Microbial Biology, Academia Sinica 71559, Taipei City, Taiwan; 3 National Mosquito-Borne Diseases Control Research Center, National Health Research Institutes 50115, Miaoli, Taiwan; Wake Forest University School of Medicine, Winston-Salem, North Carolina, USA

**Keywords:** dengue virus, glycolysis, diabetes, mitochondrial dysfunction, lactate, eIF2α, PKR

## Abstract

Dengue virus (DENV) strains with high pathogenicity and transmissibility pose significant public health challenges, especially in tropical and subtropical regions. Underlying conditions such as diabetes mellitus and renal diseases significantly increase the risk of severe dengue. The DENV-2 strain, responsible for a severe outbreak in Taiwan in 2015, exhibits enhanced pathogenicity and transmissibility in a mosquito-mouse transmission model. In this study, we demonstrated that pathogenic DENV infection leads to elevated lactate levels and hypoglycemia in mice, correlating with increased mortality in streptozotocin-induced diabetic models. In infected cells, pathogenic DENV induces rapid eIF2α phosphorylation, extensive ER membrane aggregation, disrupted calcium transfer to mitochondria, and mitochondrial dysfunction, which may contribute to excessive lactate production. Notably, inhibition of lactate production reduced viremia and mortality in mice. These findings highlight the role of metabolic dysregulation in DENV pathogenesis and provide insights into the mechanisms driving severe dengue, particularly in patients with underlying comorbidities.

**IMPORTANCE:**

DENV is a mosquito-borne virus that can cause severe illness, particularly in tropical and subtropical regions. In 2015, a strain of DENV-2 caused a major outbreak in Taiwan with high mortality rates. People with conditions like diabetes or kidney disease were more likely to develop severe dengue. In our study, we found that this highly pathogenic virus caused mice to have high levels of lactate and low blood sugar before death. In diabetic mice, the virus caused even higher death rates. The virus impairs cellular energy production by disrupting communication between the endoplasmic reticulum and mitochondria, potentially leading to excessive lactate accumulation. Blocking lactate production helped reduce viremia and death rate. These findings suggest that the virus’s impact on metabolism may play a role in severe illness, especially for people with pre-existing health issues.

## INTRODUCTION

Dengue virus (DENV), a member of the Flaviviridae family, has a single-stranded, positive-sense RNA genome and is transmitted to humans by *Aedes aegypti* and *Aedes albopictus* mosquitoes. DENV is classified into four serotypes (DENV-1 to DENV-4), each comprising multiple genotypes. Dengue outbreaks in Taiwan typically begin with imported cases during summer and fall, when *Aedes aegypti* mosquitoes are active and cease in winter as cold temperatures limit mosquito viability (1, 2). Rainfall and *Aedes aegypti* mosquito density are critical factors determining vector availability for virus transmission. The unique properties of the DENV strains, such as pathogenicity and transmissibility, may also influence the scale of outbreaks. For instance, the DENV-2 virus strain (TW2015; Cosmopolitan genotype) caused a significant outbreak in Taiwan in 2015, resulting in 43,784 confirmed dengue cases, high mortality, and severe complications (3). This virus strain demonstrates high pathogenicity in *Stat1*^-/-^ mice, which are deficient in the signal transducer and activator of transcription 1 (STAT1)—a critical mediator downstream of type I and II interferon pathways—and in AGB6 mice, which lack functional type I and II interferon receptors. The TW2015 strain also demonstrates high transmissibility between mosquitoes and vertebrate hosts (4).

Epidemiological studies of the 2015 dengue outbreak in Taiwan indicate that a high proportion of fatal cases occurred in older adults with comorbid conditions, including diabetes mellitus and chronic kidney disease (3). Diabetes has been identified as a comorbidity in dengue hemorrhagic fever and dengue shock syndrome in several retrospective studies (5–9). Diabetes is a metabolic disorder characterized by defective glucose homeostasis. Glucose serves as a primary energy source for mammalian cells, and glycolysis is a central metabolic pathway that converts glucose into pyruvate while generating ATP and NADH. Under aerobic conditions, pyruvate typically enters mitochondria to fuel the tricarboxylic acid (TCA) cycle and oxidative phosphorylation, maximizing ATP production. However, during hypoxia, mitochondrial dysfunction, or high biosynthetic demand (as in cancer or viral infection), pyruvate is predominantly converted into lactate via lactate dehydrogenase (LDH), a process referred to as anaerobic glycolysis (10). Hyperglycemia is shown to exacerbate DENV infection by facilitating viral translation (11).

Investigating whether DENV infection exacerbates metabolic stress in diabetic hosts, potentially contributing to higher mortality, is crucial. This study demonstrates that DENV TW2015 infection leads to the accumulation of lactate, resulting in enhanced mortality in diabetic mice. The underlying mechanisms were further explored.

## RESULTS

### Pathogenic DENV infection disturbs glucose metabolism in *Stat1^-/-^* mice

**Fig 1:**
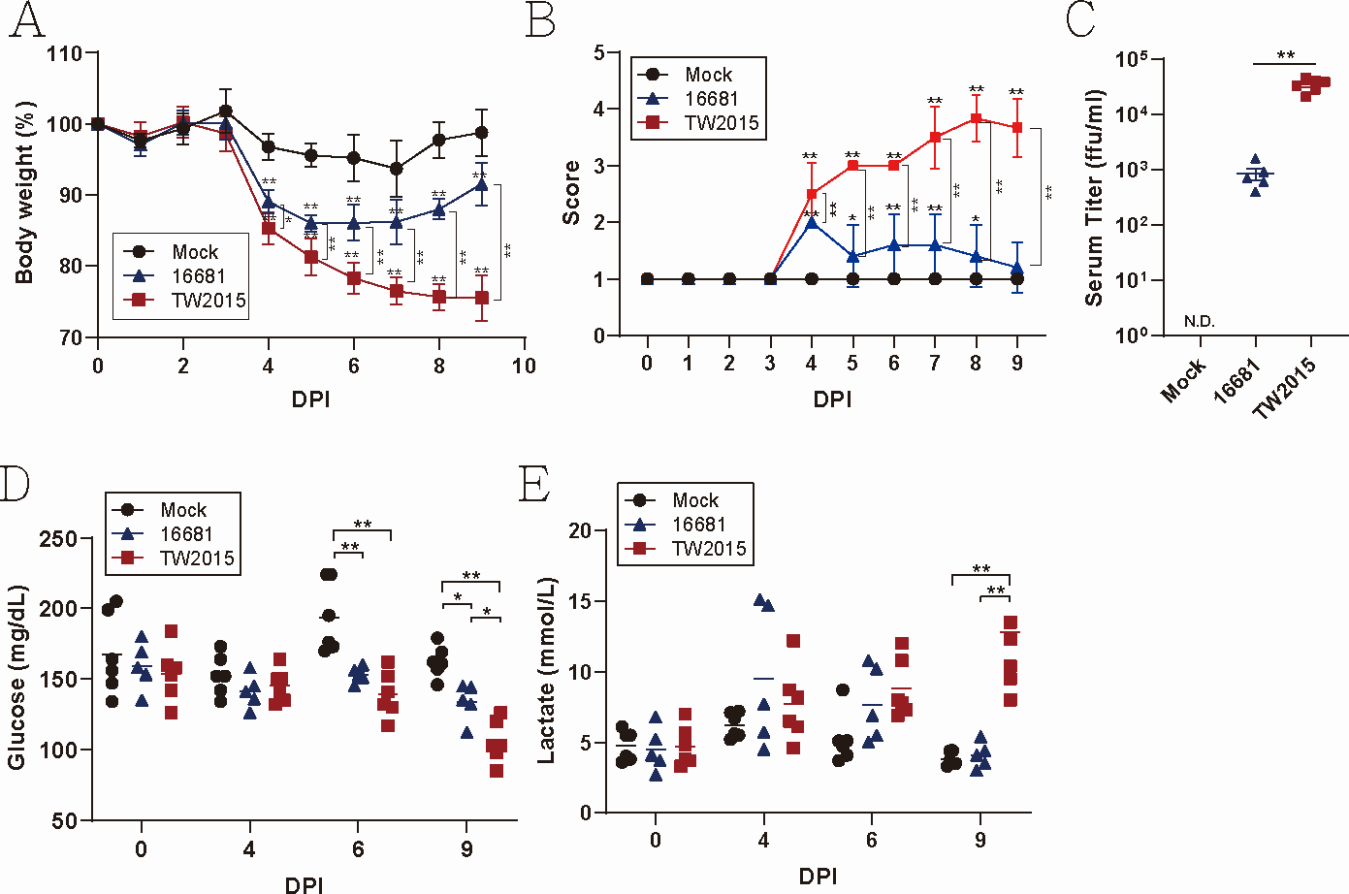
Pathogenic DENV TW2015 strain infection leads to decreased blood glucose levels and lactate accumulation. (**A**) Female *Stat1^-/-^* mice were infected with DENV-2 16681 (*n* = 5) or TW2015 (*n* = 6) strains (5 × 10⁴ PFU/mouse, intradermal). Body weight (mean ± SD) was monitored daily. (**B**) All animals were visually scored for morbidity on a score of 1 to 5 (healthy to moribund). (**C**) Blood was collected at 3 DPI, and serum virus titers were determined by a focus forming assay. Blood glucose levels (**D**) and blood lactate levels (**E**) were measured periodically. Statistical analysis was performed using the Mann–Whitney nonparametric test for (**C**), and two-way ANOVA with Tukey’s multiple comparisons test for (**A**), (**B**), (**D**), and (**E**). N.D., not detected. *P < 0.05, **P < 0.01.

During glycolysis, glucose is metabolized into pyruvate. Under aerobic conditions, pyruvate enters to participate in the mitochondria for the citric acid cycle, while under anaerobic conditions, it is converted to lactate in the cytoplasm. The TW2015 strain has been shown to exhibit higher pathogenicity *in vivo* compared to other DENV-2 strains, such as 16681 and NGC (4). To investigate the impact of DENV infection on glucose metabolism, female *Stat1^-/-^* mice were infected intradermally with DENV-2 16681 and TW2015 strains (5 × 10^4^ PFU/mouse). Subsequent monitoring included body weight, clinical score, morbidity, and levels of blood glucose and lactate. Both DENV-2 strains caused significant body weight loss, with TW2015-infected mice showing greater weight loss than those infected with the 16681 strain (Fig. 1A). 16681-infected mice showed mild symptoms (score ≤2), while TW2015-infected mice exhibited more severe signs (score ≥3), indicating higher pathogenicity of the TW2015 strain (Fig. 1B). Serum virus titers were significantly higher in TW2015-infected mice than in 16681-infected mice (Fig. 1C). Blood glucose levels in DENV-2–infected mice notably decreased, particularly at 9 DPI (Fig. 1D). Concurrently, blood lactate levels were significantly elevated in TW2015-infected mice at the same time point (Fig. 1E), suggesting that pathogenic DENV-2 infection may promote enhanced anaerobic glycolysis. Consistent with this, glycolysis-related gene activation was observed in DENV-2 TW2015–infected mice (Fig. S1). Specifically, *Hypoxia-inducible factor 1 α* (*Hif1a*), *Hexokinase 2* (*Hk2*), and *Lactate Dehydrogenase A* (*Ldha*) were upregulated in the spleen at 6 DPI. In the liver, the expression of *Glucose transporter 1* (*Glut1*), *Hexokinase 1* (Hk1), and Hk2 was also significantly increased at the same point.

**Fig 2:**
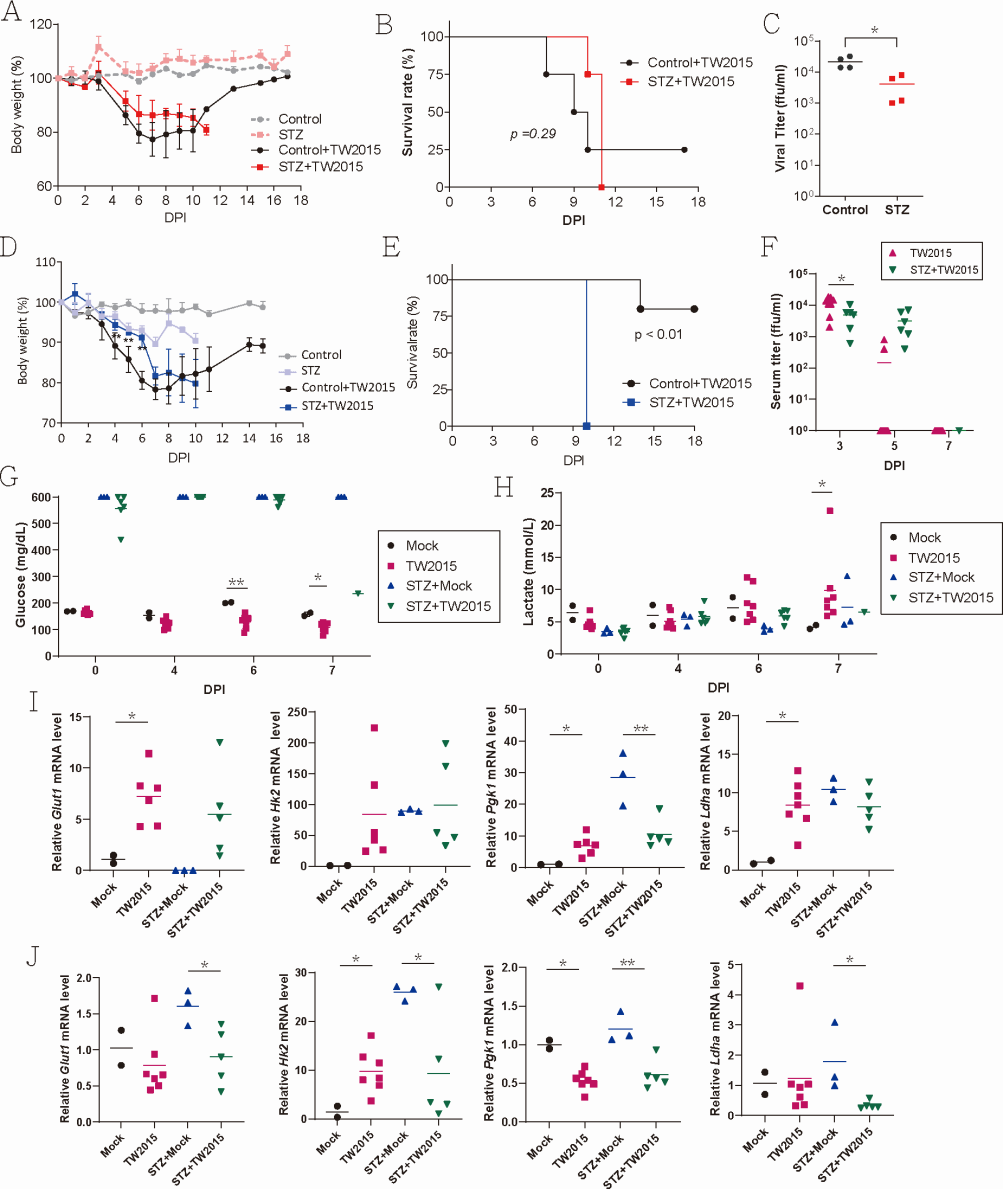
Glucose metabolism dysregulation enhances DENV-associated mortality. (**A–C**) Diabetes was induced in female *Stat1^-/-^* mice with a single intraperitoneal injection of streptozotocin (STZ, 200 mg/kg). Seven days after STZ treatment, mice (with or without STZ; *n* = 4) were infected with TW2015 virus (5 × 10⁴ PFU/mouse, intradermal). Body weight (**A**; mean ± SD), survival rate (**B**), and viral titers in blood (day 3 post-infection) (**C**) were assessed. (**D and E**) Diabetic *Stat1^-/-^* male mice induced with STZ were infected with the TW2015 virus. Body weight and survival rate were monitored (*n* = 5). (**F–J**) *Stat1^-/-^* male mice with and without STZ treatment were challenged with TW2015 virus (5 × 10⁴ PFU/mouse, intradermal; *n* = 5–6). (**F**) Serum was collected on 3, 5, and 7 DPI for virus titration. (**G**) Blood glucose and (**H**) lactate levels were measured on 4, 6, and 7 DPI. Samples with blood glucose levels exceeding the detection limit of 600 mg/dL were plotted as “600” in the figures. Four mice in the STZ-TW2015 group were found dead immediately prior to testing on 7 DPI; therefore, glucose, lactate, and serum titers were not assessed for these animals. All mice were sacrificed for tissue collection. Mouse spleens (**I**) and livers (**J**) were analyzed by RT-qPCR to measure expression of glycolysis-related genes. DPI, days post-infection. Statistical analyses: Mann–Whitney tests for (**A**), (**C**), and (**D**) (control + TW2015 vs STZ + TW2015); Log-rank (Mantel–Cox) test for (**B**) and (**E**). Unpaired *t-test* for (**F**) (control + TW2015 vs STZ + TW2015) and (**G–J**) (mock vs TW2015 infection). ns, no significance. *P* < 0.05 (*), *P* < 0.01 (**).

Given the increased risk of severe dengue and mortality associated with diabetes mellitus, the impact of DENV infection was further examined in diabetic mice. Female *Stat1^-/-^* mice were administered a single intraperitoneal injection of streptozotocin (STZ, 200 mg/kg) to induce pancreatic islet β-cell destruction (12). Blood glucose levels in STZ-treated mice reached approximately 300 mg/dL, nearly double those of control mice (average 136 mg/dL) (Fig. S2A). Subsequently, the mice were infected with the TW2015 virus (5 × 10⁴ PFU/mouse, intradermal). Despite experiencing slightly less severe body weight loss than controls, STZ-treated mice exhibited a 100% mortality rate, in contrast to the 50% mortality rate observed in control mice (Fig. 2A and B). Serum virus titers on day 3 post-infection were reduced in STZ-treated mice (Fig. 2C). Male mice, known to be more sensitive to STZ treatment, exhibited blood glucose levels reaching up to 600 mg/dL and reduced blood lactate levels within a week (Fig. S2B). Following DENV-2 infection, STZ-treated male mice demonstrated a 100% mortality rate compared to a 25% mortality rate in control mice (Fig. 2D and E).

When the kinetics of virus titer was examined, TW2015-infected male mice exhibited significantly higher serum viral loads compared to the STZ + TW2015 group at 3 DPI (Fig. 2F). By 5 DPI, more than half of the TW2015-infected mice had undetectable viral titers, whereas the STZ + TW2015 group maintained relatively high levels (ranging from 10² to 10⁴ ffu/mL), suggesting that diabetic mice exhibit delayed viral replication kinetics. In the STZ + TW2015 group, blood glucose levels remained consistently elevated throughout the course of infection, except during the moribund state observed at 7 DPI (Fig. 2G). Additionally, lactate levels in STZ-treated mice remained low, even after DENV-2 infection (Fig. 2H), indicating impaired glycolytic flux under diabetic conditions.

To explore potential mechanisms associated with the high mortality observed in TW2015-infected diabetic mice, gene expression in mouse organs at 7 DPI was analyzed using real-time RT-qPCR. Several glycolysis-related genes were significantly upregulated in STZ-treated mice. In particular, *Hk2*, *Pgk1* (phosphoglycerate kinase 1), and *Ldha* expression were elevated in the spleen, while *Glut1* (glucose transporter 1), *Hk2*, and *Ldha* were upregulated in the liver. Notably, this elevated expression was not further enhanced by DENV-2 TW2015 infection in the STZ-treated mice but was elevated in control mice after TW2015 infection (Fig. 2I and J). Impaired glucose regulation may contribute to the increased mortality observed in DENV-2-infected STZ-treated mice.

### Highly pathogenic DENV-2 virus infection increases lactate production in cells

**Fig 3:**
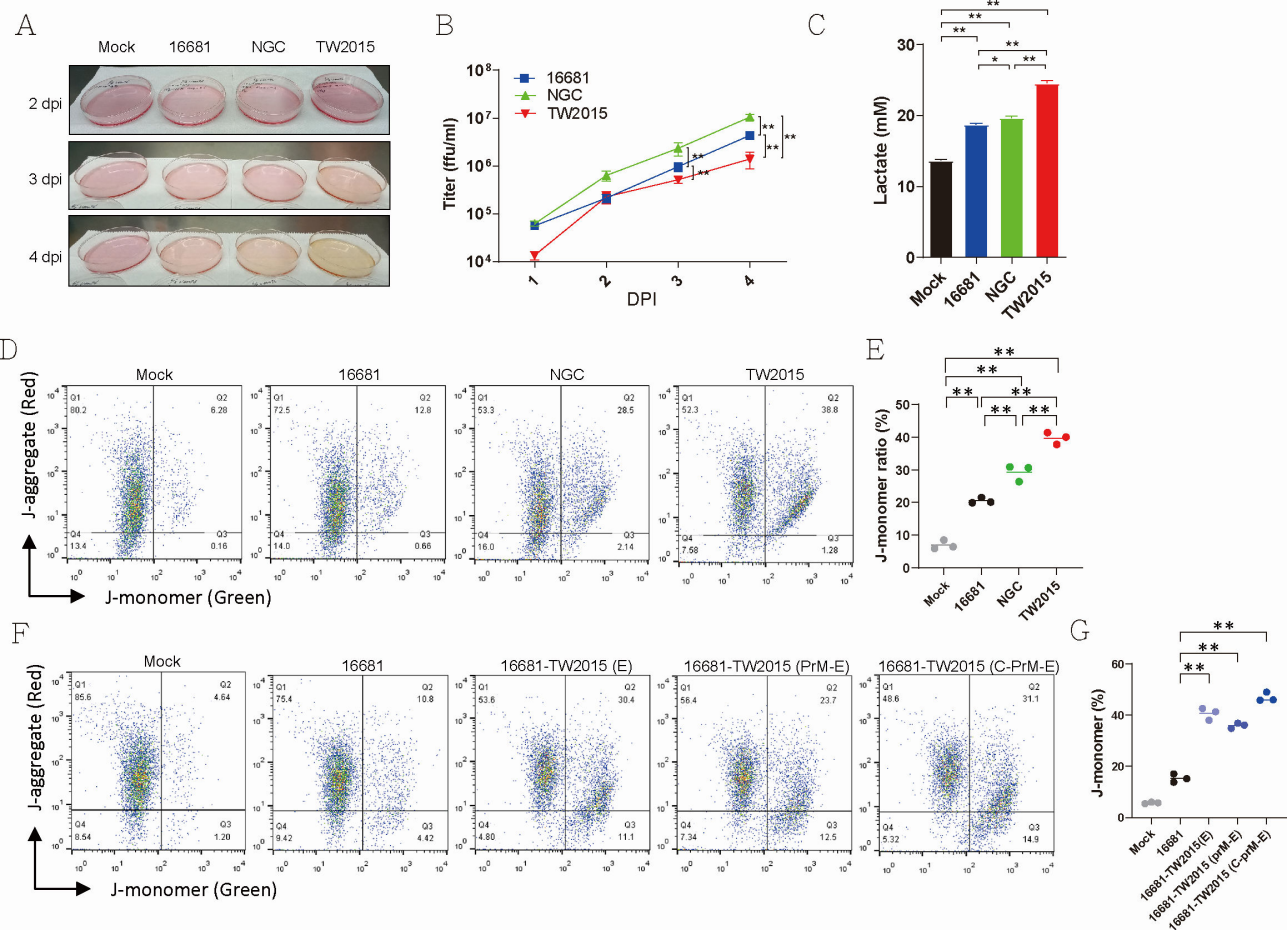
Pathogenic DENV infection induced lactate elevation and mitochondrial dysfunction. (**A**) Vero cells were infected with DENV-2 strains (16681, NGC, and TW2015; m.o.i. = 0.1). Color changes in the culture medium were documented on 4 dpi. (**B**) Virus titers were assessed from 1 to 4 DPI (*n* = 3). Statistical analysis was performed using two-way ANOVA with Tukey’s multiple comparisons test; **P < 0.01. (**C**) Lactate concentration in the culture medium was quantified at 4 DPI (*n* = 3). Statistical significance was determined using one-way ANOVA; **P < 0.01. (**D and E**) Vero cells infected with DENV-2 strains were analyzed on 3 DPI for mitochondrial activity using JC-1 dye and flow cytometry. Representative results are shown in (**D**), and the percentage of JC-1 monomer-positive cells is quantified in (**E**) (*n* = 3). Statistical significance was determined using one-way ANOVA; **P < 0.01. (**F and G**) Vero cells were infected with 16681-based recombinant viruses containing the E, prM-E, or C-prM-E fragments from the TW2015 strain. Mitochondrial activity at 3 DPI was assessed via JC-1 staining (*n* = 3). Statistical significance was determined using one-way ANOVA; **P < 0.01.

The pathogenic DENV-2 TW2015 virus can penetrate extralymphatic organs, such as the lungs and intestines in mice (4). Lactate induction in DENV-infected *Stat1^-/-^* mice may arise directly from infected cells or indirectly due to hypoxia associated with pulmonary dysfunction. To investigate this, the potential of the pathogenic DENV-2 virus to induce lactate production was further examined. When DENV-2 viruses were amplified in Vero cells, the culture medium of TW2015-infected cells became distinctly acidic by day 4 post-infection, as evidenced by the phenol red color change in the medium (Fig. 3A). Direct pH measurements further confirmed a significant decrease in the pH of the TW2015-infected cell culture medium on days 3 and 4 post-infection (Fig. S3). NGC-infected cells exhibited a slight pH decrease on day 4 post-infection, while 16681-infected cells showed no significant pH changes compared to mock-infected cells. All tested strains replicated efficiently in Vero cells; however, TW2015-infected cells showed relatively lower titers compared to the other two strains at 3 and 4 DPI (Fig. 3B). Since lactate, a byproduct of anaerobic glycolysis, can lower pH in culture medium, lactate levels in the medium of DENV-2-infected cells were measured on day 4. As shown in Fig. 3C, DENV infections in Vero cells led to lactate accumulation compared to mock infections, with TW2015-infected cells secreting a significantly greater amount of lactic acid into the medium. This pH reduction associated with the TW2015 virus infection was also observed in Huh7 hepatoma cells (Fig. S4). Overall, these findings suggest that pathogenic DENV-2 viruses alter glucose metabolism in infected cells.

### Pathogenic DENV-2 infection disrupts mitochondrial function

DENV infection induces mitochondrial dysfunction (13–15). To determine whether mitochondrial dysfunction contributes to lactic acid production from anaerobic glycolysis, JC-1 dye staining was used to assess mitochondrial membrane potential in DENV-2–infected cells. In healthy mitochondria, the cell-permeable JC-1 dye forms red fluorescent J-aggregates, while in dysfunctional mitochondria, it remains as green fluorescent J-monomers. Fewer than 10% of mock-infected cells showed mitochondrial dysfunction. In contrast, significant mitochondrial dysfunction was observed in DENV-2–infected cells by day 3 post-infection (Fig. 3D and E). The highly pathogenic TW2015 strain induced markedly greater mitochondrial dysfunction in Vero cells compared to the 16681 and NGC strains. Given the critical role of the prM-E region in TW2015 pathogenesis (4), Vero cells were infected with recombinant 16681 viruses carrying C-prM-E, prM-E, or E segments from TW2015 and were subjected to JC-1 staining. Higher levels of mitochondrial dysfunction were detected in cells infected with 16681-TW2015(E), 16681-TW2015(prM-E), and 16681-TW2015(C-prM-E) viruses compared to those infected with 16681 virus (Fig. 3F and G). Mitochondrial dysfunction induced by highly pathogenic DENV-2 strains, TW2015 and 16681-TW2015(C-PrM-E), may initiate as early as day 2 post-infection (Fig. S5A). Additionally, TW2015 virus infection also induced mitochondrial dysfunction in Huh7 cells (Fig. S5B). These results suggest that lactic acid production may be driven by mitochondrial dysfunction triggered by pathogenic DENV infection.

### Pathogenic DENV-2 virus infection impairs ER–mitochondria communication

**Fig 4:**
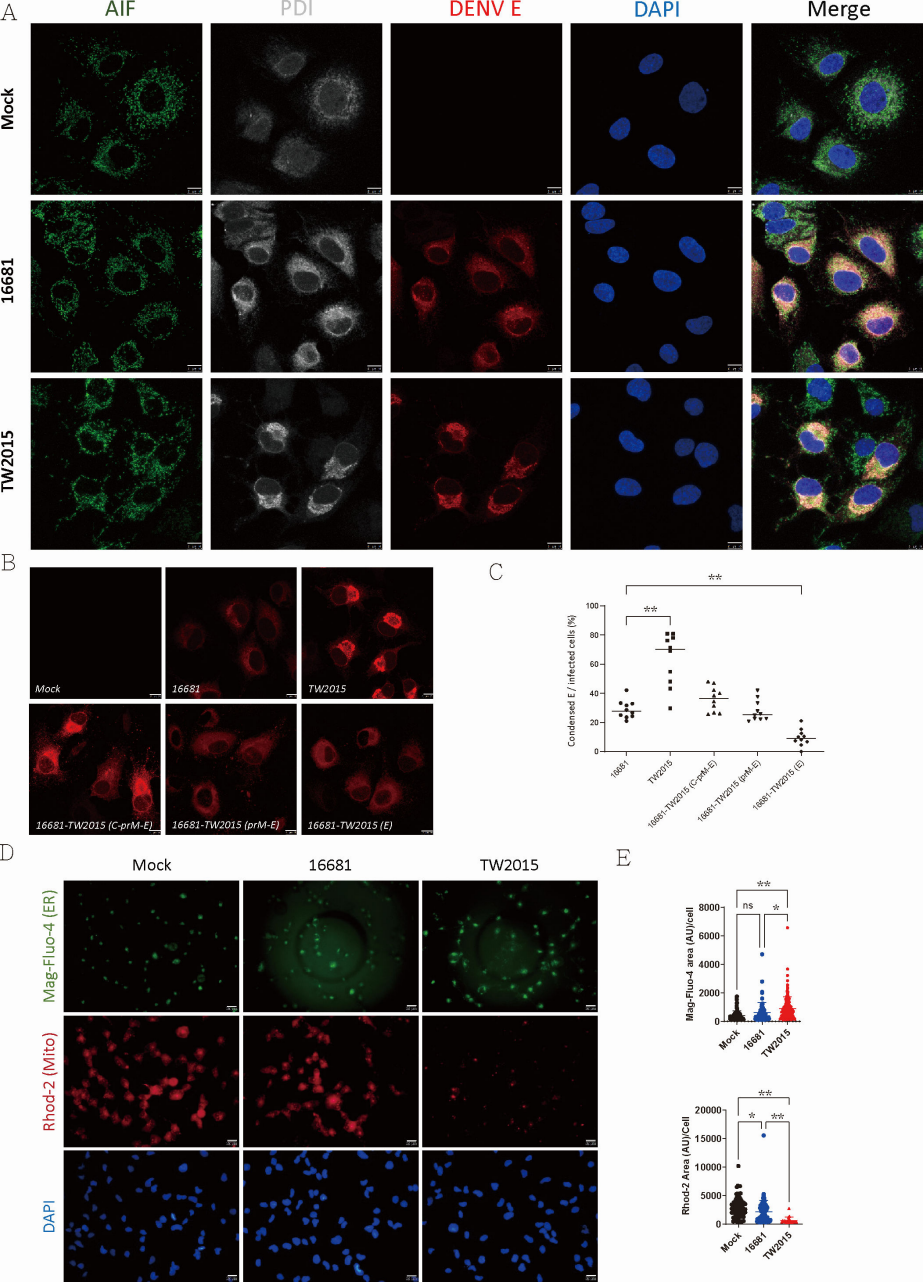
ER structural alterations induced by pathogenic DENV infection. (**A**) Huh7 cells infected with the TW2015 virus for 48 h were stained with antibodies against DENV E protein, AIF (a mitochondrial marker), and PDI (an ER marker). Images were captured using a confocal microscope. Scale bar: 10 µm. (**B**) Cells infected with 16681 recombinant viruses carrying the E, prM-E, or C-prM-E fragments from the TW2015 virus were stained with an anti-E antibody. Images of cells infected with 16681-TW2015(E) and 16681-TW2015(prM-E) were captured with increased exposure times. Scale bar: 10 µm. (**C**) Huh7 cells infected with DENV-2 strains were stained with an anti-E antibody. Images of randomly selected fields were captured (*n* = 10). The percentage of infected cells exhibiting E protein condensation localized to one side was quantified. Statistical significance was determined using one-way ANOVA; **P < 0.01. (**D**) Free Ca²^+^ concentrations in the ER lumen and mitochondria of DENV-2–infected cells (moi = 0.5; 2 DPI) were traced by staining (2 µM, 30 min) with Mag-Fluo-4 and Rhod-2, respectively. Scale bar: 20 µm. (**E**) The fluorescent signal area of Mag-Fluo-4 (*n* = 68–133) and Rhod-2 (*n* = 20–95) was quantified on a per-cell basis. Statistical significance was determined using one-way ANOVA; *P < 0.05, *P* < 0.01.

DENV replication and assembly primarily occur in the endoplasmic reticulum (ER). To explore the relationship between TW2015 virus infection, mitochondrial dysfunction, and metabolic alterations, infected Huh7 cells were stained with antibodies for the DENV E protein and organelle markers for mitochondria (AIF) and ER (PDI). As shown in Fig. 4A, E protein expression in DENV-2–infected cells predominantly co-localized with the ER marker. In TW2015-infected cells, the ER structure appeared highly condensed and aggregated to one side of the cytoplasm, a feature less pronounced in cells infected with the 16681 strain. The mitochondrial staining patterns in DENV-2–infected cells showed no significant changes in shape or length.

The distribution of the E protein in cells infected with 16681-TW2015(E), 16681-TW2015(prM-E), and 16681-TW2015(C-PrM-E) viruses was analyzed at day 2 post-infection (Fig. 4B; Fig. S6). The E protein distribution in 16681-TW2015(C-PrM-E)-infected cells closely resembled that of TW2015-infected cells. E protein expression levels in cells infected with 16681-TW2015(E) and 16681-TW2015(prM-E) were relatively low, requiring extended exposure times for confocal microscopy imaging in Fig. 4B. The E protein distribution patterns for these two recombinant viruses were similar to the E protein pattern of the 16681 viruses. Image quantification revealed that approximately 70% of TW2015-infected cells exhibited condensed E protein distribution, compared to less than 30% in 16681-infected cells (Fig. 4C). These results suggest that pathogenic DENV-2 infection induces an ER structural alteration where the E protein is concentrated.

Ca^2+^ is primarily stored in the ER and transported to mitochondria to regulate ATP production (16). DENV infection is shown to reduce ER–mitochondria contact site and regulate respiration (17). The potential disruption of Ca²^+^ transport between the ER and mitochondria in TW2015-infected cells was further investigated. DENV-2–infected cells were stained with Mag-Fluo-4 AM (18) and Rhod-2 AM (19) to trace the Ca^2+^ levels in the ER and mitochondria, respectively. The ER Ca²^+^ levels in TW2015-infected cells were obviously increased at 2 DPI (Fig. 4D and E). In contrast, Rhod-2 signals indicating mitochondrial Ca²^+^ levels were significantly lower in TW2015-infected cells compared to mock or 16681-infected cells. These results indicate that TW2015 infection disrupts Ca²^+^ transport from the ER to mitochondria. In summary, pathogenic DENV-2 infection induces ER alterations and disrupts ER–mitochondria Ca²^+^ transport, both of which may contribute to mitochondrial dysfunction.

### Pathogenic DENV-2 infection induces ER membrane aggregation

**Fig 5:**
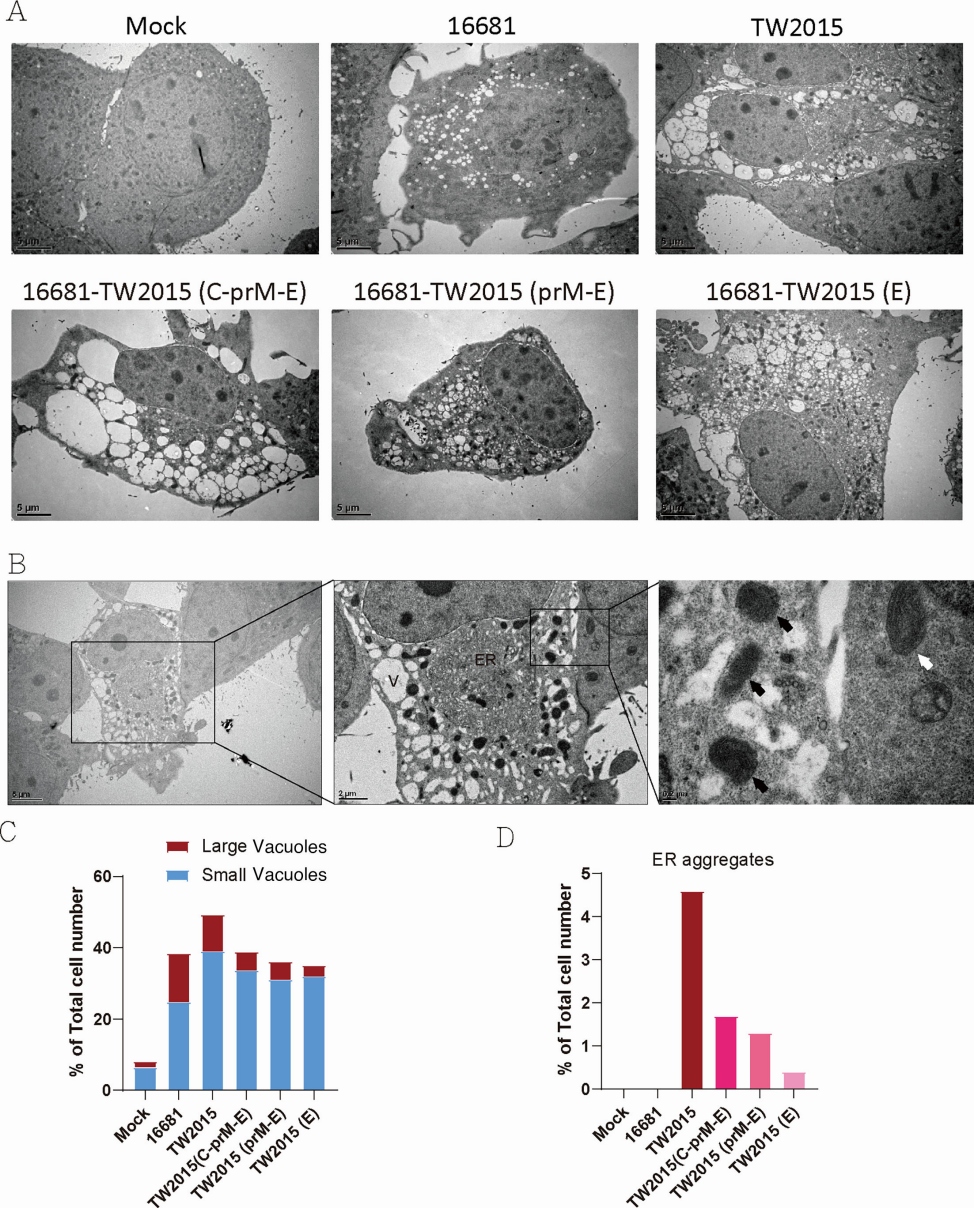
Pathogenic DENV infection induces vacuole accumulation and ER membrane aggregates. (**A**) Huh7 cells infected with DENV-2 at 2 DPI were analyzed using transmission electron microscopy to observe structural changes. Scale bar: 5 µm. (**B**) Enlarged views of mitochondria in TW2015-infected cells (black arrows) and adjacent uninfected cells (white arrow) highlight differences in mitochondrial morphology. ER, aggregated ER; V, vacuole. Scale bar: 5–0.2 μm. (**C**) For quantification, approximately 50 images per sample grid (field of view: 80 µm × 80 µm) were captured under different infection conditions using a Borries K004 STEM at 30 kV accelerating voltage. A total of 500–600 cells were analyzed, and the percentages of cells containing small or large vacuoles were determined. Cells exhibiting ER aggregation were quantified separately, as shown in panel (**D**) Scale bar: 10 µm. Figure S7 shows representative electron microscopy images of DENV-infected cells exhibiting vacuolar structures and ER morphological alterations.

Membrane alterations are commonly associated with virus replication and assembly (20, 21). DENV infection induces ER-derived membranous structures containing nonstructural proteins for RNA replication (20, 22). To investigate the structural changes induced by pathogenic DENV-2 infection, Huh7 cells infected with 16681, TW2015, and recombinant viruses were analyzed by transmission electron microscopy at 48 h post-infection. As shown in Fig. 5A, 16681-infected cells exhibited vacuoles compared to mock-infected cells. In contrast, TW2015-infected cells displayed numerous large vacuoles, with aggregated ER membranes localized at one side near the nuclei (Fig. 5A). Additionally, mitochondria in TW2015-infected cells appeared condensed, as highlighted in the enlarged image in Fig. 5B, with most mitochondria excluded from the ER aggregates. Large vacuoles were also observed in 16681-TW2015(C-prM-E)-infected cells, although the ER aggregates were relatively dispersed (Fig. 5A). Cells infected with 16681-TW2015(prM-E) and 16681-TW2015(E) did not exhibit pronounced ER condensation but still contained numerous vacuoles. Quantification of ultrastructural changes under different infection conditions was performed using scanning TEM (STEM) imaging technology by Borries (Singapore) (Fig. S7). TW2015-infected cells exhibited a higher percentage of cells with small and large vacuoles (49.4%) compared to mock-infected controls (8.2%) and 16681-infected cells (38.5%). Recombinant viruses 16681-TW2015 (C-prM-E), (prM-E), and (E) induced moderate levels of vacuole formation (35.1%–38.9%; Fig. 5C). Regarding ER aggregation, only the TW2015 strain induced prominent ER condensation (4.6%), with lower frequencies observed in 16681-TW2015 (C-prM-E) (1.7%) and (prM-E) (1.3%) (Fig. 5D). Minimal or no ER aggregation was observed in the mock, 16681, and 16681-TW2015 (E) groups. The relationship between the accumulation of autophagosome- or lysosome-like vacuoles and mitochondrial dysfunction in the context of pathogenic viral infection remains to be further investigated.

### Pathogenic DENV-2 infection stimulates eIF2α phosphorylation-associated stress response

**Fig 6:**
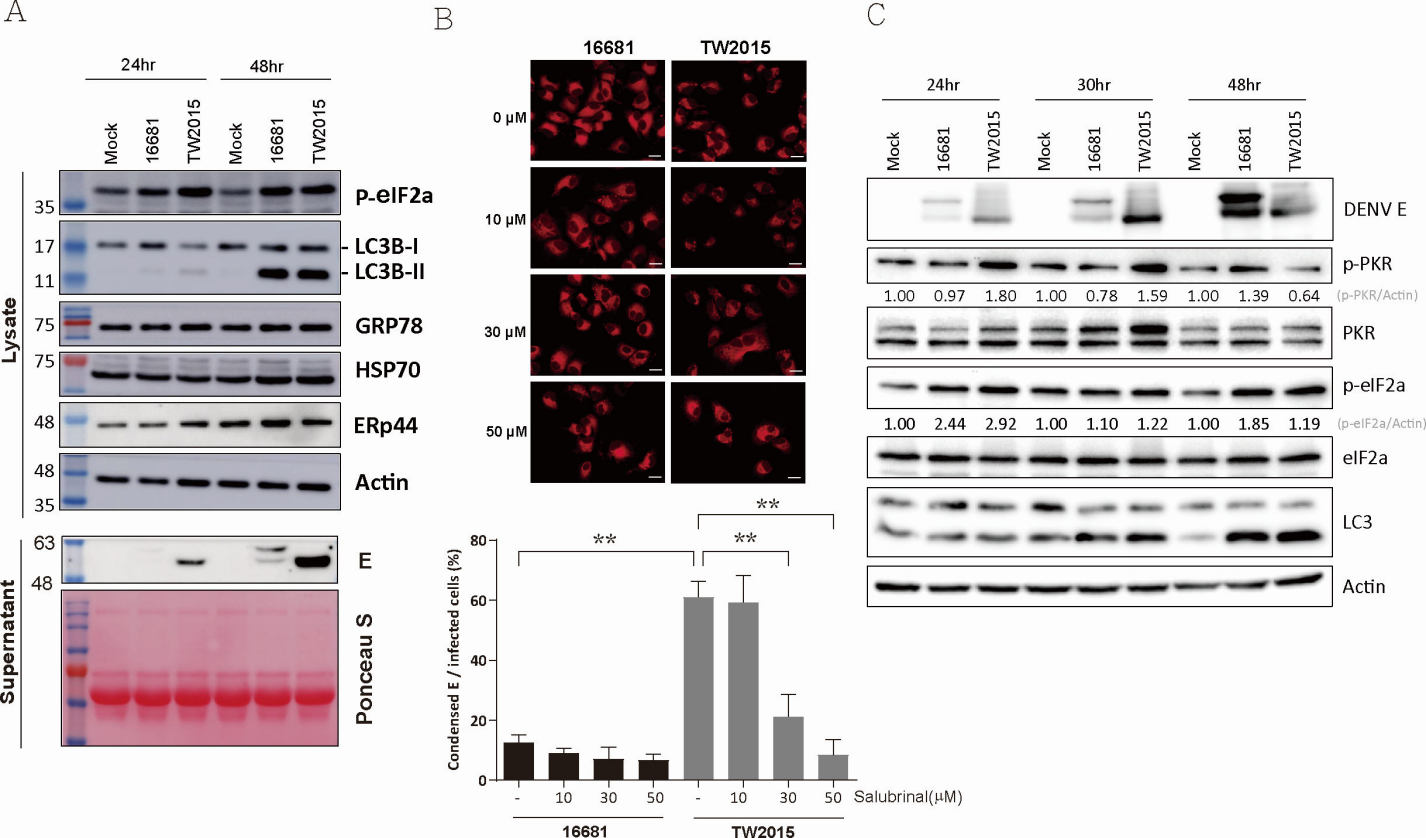
PRK activation and eIF2α phosphorylation are elevated in pathogenic DENV-infected cells. (**A**) Huh7 cells infected with DENV-2 viruses at 24 and 48 h post-infection were subjected to immunoblotting to detect ER stress-related markers (phospho-eIF2α, GRP78, HSP70, and ERp44) and the autophagy marker LC3 in cell lysate, as well as the DENV E protein in culture supernatants. Ponceau S staining served as a loading control. (**B**) Huh7 cells infected with DENV-2 for 24 h were treated with salubrinal, an inhibitor of eIF2α dephosphorylation, for an additional 24 h. E protein expression patterns were examined by immunostaining, and the percentage of cells showing condensed E protein expression was quantified (*n* = 10). Statistical significance was determined using one-way ANOVA; *P < 0.01. Scale bar: 20 µm. (**C**) Phospho-PKR and phospho-eIF2α kinetics in DENV-2–infected Huh7 cells were analyzed by immunoblotting and quantified using ImageJ software.

RNA virus infection can induce ER stress and activate the unfolded protein response (UPR), leading to an autophagic response (23–25). It is plausible that ER stress contributes to the ER aggregation observed in pathological DENV infection. To investigate whether the ER stress response is differentially activated by pathogenic DENV, the protein expression levels of ER stress regulatory proteins (GRP78, HSP70, and ERp44), the eIF2α phosphorylation level, and the autophagy marker LC3 were evaluated in DENV-2–infected cells. In Fig. 6A, the eIF2α phosphorylation level increased in TW2015-infected cells at both 24 and 48 h post-infection, earlier than in 16681-infected cells. Autophagy was activated by both TW2015 and 16681 virus infections, as indicated by the elevated LC3B-II levels at 48 h post-infection. The protein expression levels of GRP78, HSP70, and ERp44 did not show significant changes in DENV-2–infected cells. Phosphorylation of eIF2α leads to the inhibition of protein translation as a cellular mechanism to combat stress or viral invasion. Although the infections were carried out using the same multiplicity of infection (MOI = 1), virus production in the culture supernatant was higher in TW2015-infected cells compared to those infected with the 16681 strain (Fig. 6A).

To investigate whether disturbance of eIF2α phosphorylation affects ER aggregation induced by TW2015 infection, salubrinal, an inhibitor of eIF2α dephosphorylation that protects cells from ER stress (26), was added during DENV-2 infection. Salubrinal treatment in Huh7 cells was well tolerated, as evidenced by the absence of significant cytotoxic effects at the concentrations used (Fig. S8A). As indicated by the condensed DENV E protein localization (Fig. 6B), ER aggregation induced by TW2015 infection significantly decreased with salubrinal treatment. Virus replication and eIF2α phosphorylation were also reduced upon salubrinal treatment (Fig. S8). The results suggest that ER aggregation may be due to continuous eIF2α phosphorylation/dephosphorylation or high levels of viral protein accumulation in the ER.

During ER stress, the unfolded protein response (UPR) is activated, leading to eIF2α phosphorylation by PKR-like ER kinase (PERK) (27). To evaluate whether the UPR serves as a critical upstream event for ER alterations induced by TW2015 virus infection, ER chemical chaperones tauroursodeoxycholic acid (TUDCA) and 4-phenylbutyric acid (4-PBA) (28) were used during DENV-2 infection. Treatment with these chemical chaperones did not affect the aggregation pattern of the TW2015 E protein (Fig. S9 and S10), indicating that the UPR may not be essential for ER aggregation caused by pathogenic DENV-2 infection. eIF2α phosphorylation can also be triggered by various stress responses, including virus infection-associated PKR activation, to modulate translation (29). Hence, PKR activation during DENV-2 infection was assessed via immunoblotting for phosphorylated PKR (Fig. 6C). Elevated levels of PKR phosphorylation, correlating with eIF2α phosphorylation, were observed at early time points (24 and 30 h post-infection) in TW2015-infected cells compared to 16681-infected cells. These findings suggest that PKR activation precedes eIF2α phosphorylation during pathogenic DENV-2 infection.

### Lactate production enhances DENV-2 virus penetration in mice

**Fig 7:**
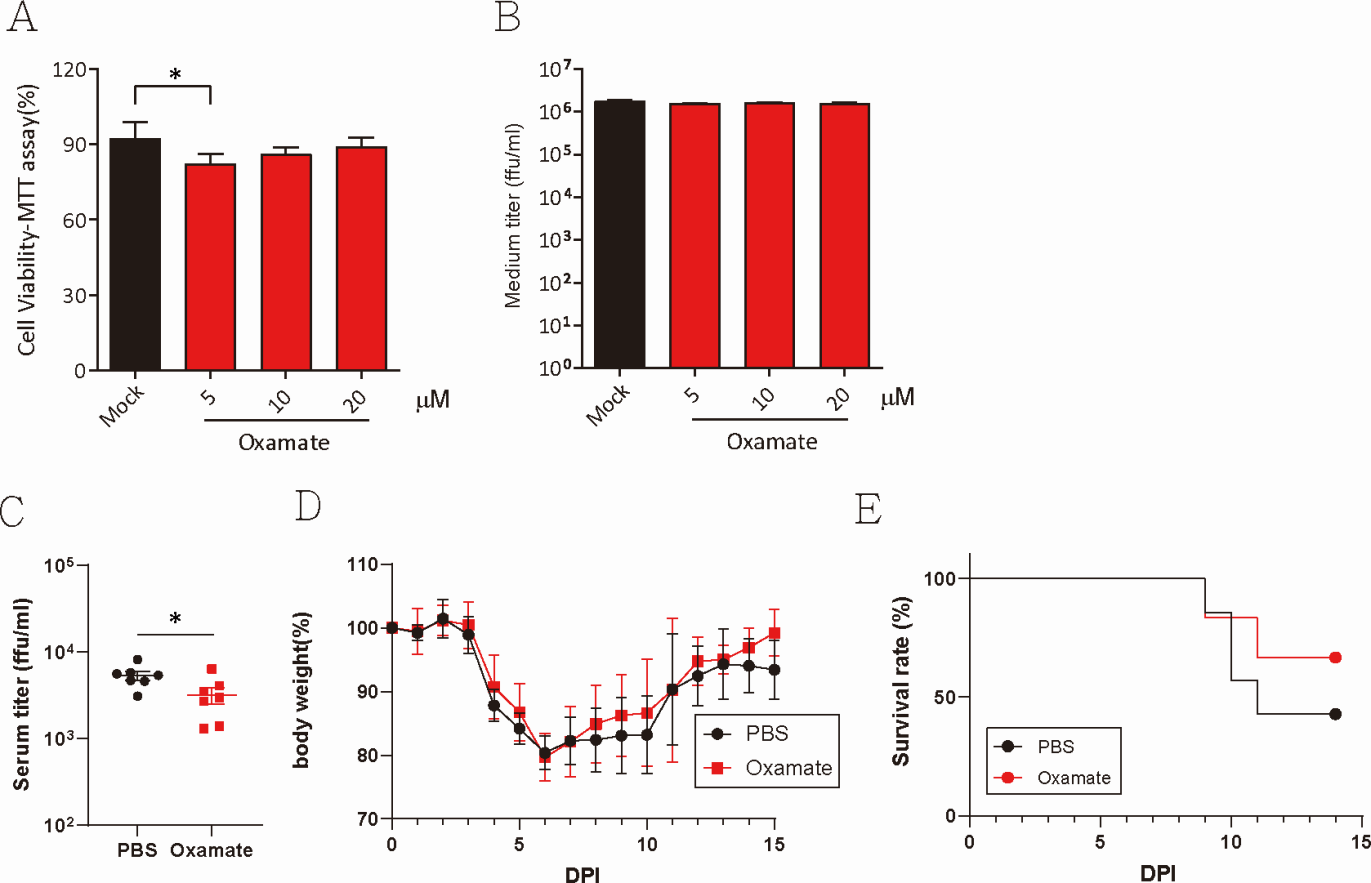
Inhibiting lactic acid production suppresses pathogenic DENV infection in mice. (**A**) Huh7 cells treated with LDH inhibitor, oxamate, for 48 h were assessed for cell viability using the MTT assay (*n* = 4–5). Statistical significance was determined using one-way ANOVA; *P < 0.05. (**B**) Virus titers in Huh7 cells infected with DENV-2 (2 DPI) in the presence of oxamate were quantified (*n* = 4–5). (**C–E**) Female *Stat1^-/-^* mice (*n* = 7) were treated with oxamate (0.75 g/kg, i.p.) or vehicle control (PBS) 2 h prior to infection and once daily thereafter. Virus titers in blood collected at 3 DPI were measured (**C**). Statistical significance was determined using one-way ANOVA; *P < 0.05. Mouse body weight (**D**) and survival rate (**E**) were monitored daily.

With mitochondrial dysfunction, TW2015 virus-infected cells might rely on anaerobic glycolysis for energy production. Lactate dehydrogenase (LDH) converts pyruvate from glycolysis to lactate. Whether lactate accumulation affects virus replication *in vitro* was tested using LDH inhibitor sodium oxamate during virus infection. While sodium oxamate treatment slightly reduced cell viability, it had no significant effect on virus production in Huh7 cells (Fig. 7A and B). To assess the role of lactate secretion during pathogenic virus infection *in vivo*, *Stat ^-/-^* mice were treated with sodium oxamate (0.75 g/kg) (30) 2 h prior to TW2015 infection and then daily post-infection. As shown in Fig. 7C, serum virus titers at 3 DPI were reduced in the sodium oxamate-treated group. Although body weight did not change significantly (Fig. 7D), sodium oxamate treatment (*n* = 6) reduced mortality from 57.1% in the PBS-treated group (*n* = 7) to 33% (Fig. 7E), suggesting that lactate overproduction may partially exacerbate DENV-induced pathology.

## DISCUSSION

**Fig 8:**
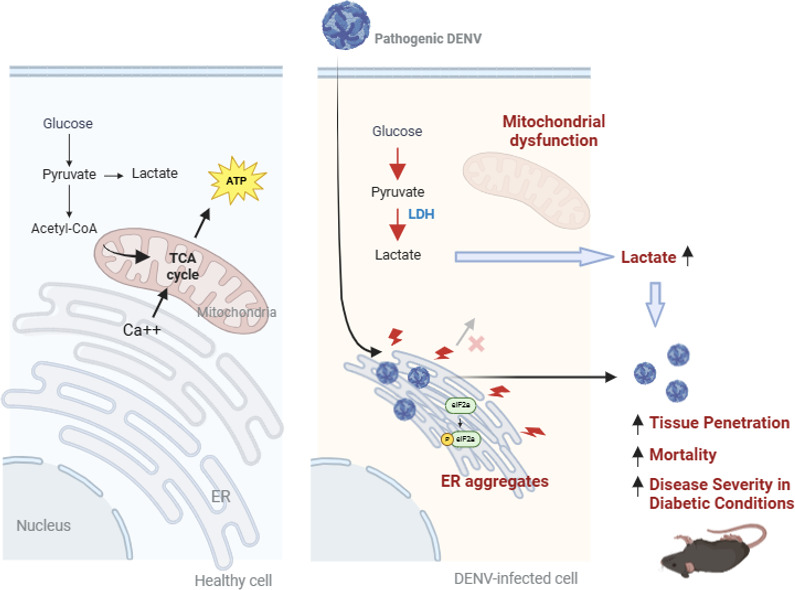
Alterations in glucose metabolism during pathogenic DENV infection. The figure is created with BioRender.com.

The study identified that infection with highly pathogenic DENV-2 induces lactate accumulation both *in vivo* and *in vitro* and further examined the intracellular alterations driving the switch to anaerobic glycolysis. In healthy cells, the ER and mitochondria are connected through contact sites that facilitate the exchange of lipids and Ca²^+^, ensuring proper regulation of energy production and glucose homeostasis (31). Mitochondrial Ca²^+^ levels regulate dehydrogenase activity in the tricarboxylic acid (TCA) cycle, directly impacting ATP production (32, 33). Our findings show that pathogenic DENV-2 infection results in ER aggregation, impaired Ca²^+^ transfer from the ER to mitochondria, and mitochondrial dysfunction. PKR activation and eIF2α phosphorylation are dominant ER stress responses induced in the infected cells. All these alterations may drive energy production to anaerobic glycolysis and lead to lactate accumulation in infected cells (Fig. 8). The accumulation of lactate from anaerobic glycolysis worsens disease severity *in vivo*, as inhibition of lactate production in infected mice reduces both viral titers and mortality rates. Moreover, mice under diabetic conditions exhibit heightened sensitivity to DENV-2 infection, with increased mortality, suggesting that functional glycolysis is essential for survival during infection. This study provides insights into why diabetes mellitus significantly increases the risk of mortality in severe dengue cases.

The contact sites between the ER and mitochondria, known as mitochondria-associated membranes (MAMs), are essential for energy production and various cellular functions. Ca²^+^ transport from the ER to mitochondria is facilitated by a channel complex involving inositol 1,4,5-trisphosphate receptors (IP3Rs) on the ER membrane, voltage-dependent anion channel 1 (VDAC1) on the outer mitochondrial membrane, and glucose-regulated protein 75 (GRP75) (16, 34). Disruption of MAMs suppresses Ca²^+^ transfer from the ER to mitochondria, leading to reduced ATP production. During DENV-2 infection, the protein expression levels of IP3R1, GRP75, and VDAC1 remained unchanged (data not shown). In TW2015-infected cells, the ER membrane becomes tightly aggregated, reducing its interaction with mitochondria. This may impair the formation of MAMs and limit the assembly of Ca²^+^ transport complexes. Consistently, Freppel et al. reported that DENV infection reduces ER–mitochondria contact sites and disrupts mitochondrial respiratory metabolism at 48 and 72 h post-infection (17).

DENV infection is known to induce the formation of ER-derived convoluted membranous structures within 24 hours post-infection. These structures, typically 2–3 µm in size, are densely packed with several nonstructural proteins and double-stranded RNAs (20, 22). In contrast, ER aggregates observed in TW2015-infected cells at 48 h post-infection range from 7 to 12 µm in size, suggesting a more extensive ER rearrangement likely driven by infection-induced cellular stress at later stages of infection. To further elucidate the dynamics of this process, immunostaining coupled with quantitative imaging analyses will be required to characterize the kinetics of ER aggregate formation in DENV-infected cells throughout the course of infection.

Comorbidities such as cardiovascular disease, stroke, diabetes, respiratory disease, and renal disease are significant risk factors for severe dengue and increased mortality. During the 2015 dengue outbreak in Taiwan, diabetes mellitus and chronic kidney disease were notably critical underlying conditions in dengue hemorrhagic cases. The TW2015 virus-infected cells exhibited mitochondrial dysfunction, relying on anaerobic glycolysis for energy production, which may exacerbate glucose dysregulation in dengue patients with diabetes. Furthermore, excessive lactic acid accumulation might be inadequately cleared in patients with renal dysfunction. These findings provide insight into how these comorbidities can aggravate dengue infection symptoms. In our study, the STZ model was used to induce β-cell damage, thereby modeling type 1 diabetes rather than type 2 diabetes, which is a more common comorbidity in dengue patients. To address this limitation, future studies should validate the effects of pathogenic DENV infection in type 2 diabetes models, such as genetic models or high-fat diet–induced insulin resistance models.

Our findings reveal that excessive lactate accumulation contributes to the pathogenesis of virulent DENV infection. However, the mechanisms by which lactate influences DENV pathogenesis remain unclear. Beyond serving as a glycolysis byproduct, lactate plays a regulatory role in various biological processes, including antiviral responses. For example, lactate can bind to MAVS, suppressing intracellular viral RNA sensing and type I interferon production (35, 36). Elevated lactate levels are also known to create an immunosuppressive environment by modulating immune cell function, as seen during tumor progression (37, 38). Further investigation is needed to determine whether lactate elevation caused by pathogenic DENV infection suppresses host immunity and impairs viral clearance.

A difference in E protein size between the TW2015 and 16681 strains was observed by immunoblotting (Fig. 6), suggesting that variations in E protein amino acid composition or post-translational modifications—such as glycosylation—may contribute to this discrepancy. To investigate this possibility, we conducted amino acid sequence analysis, which confirmed that the major N-glycosylation sites are conserved between the TW2015 and 16681 strains. Furthermore, PNGase F digestion did not suggest any significant differences in N-glycosylation between the strains. Whether other post-translational modifications contribute to the differences in E protein size or influence the pathogenic properties of these strains remains to be determined and warrants further investigation.

## MATERIALS AND METHODS

### Viruses and cells

NGC and 16681 viruses were obtained from Dr. Andrew Yueh (NHRI, Taiwan). The TW2015 virus was obtained from the Centers for Disease Control. Recombinant 16681 viruses containing various TW2015 fragments (E, prM-E, or C-prM-E) were generated and described previously (4). All virus strains were amplified in Vero 76 cells and titrated by colorimetric focus-forming assay (4). Vero cells were cultured with 1× Dulbecco’s modified Eagle medium (DMEM) supplemented with 2% FBS and 1% antibiotic-antimycotic at 37°C. Huh7 cells were cultured with DMEM supplemented with 10% FBS and 1% antibiotic-antimycotic.

### Mice

*Stat1^-/-^* (39) mice were bred and maintained at the Laboratory Animal Center of NHRI. For virus infection, TW2015 and 16681 virus (5 × 10^4^ in 200 µL) were inoculated intradermally at four sites on the upper back of mice at 8–12 weeks of age (40). Morbidity scoring was based on a scale of 1 to 5 described previously (41, 42): 1, healthy; 2, mild signs of lethargy; 3, lethargy, ruffled fur, and hunched posture; 4, lethargy, ruffled fur, hunched posture, and decreased mobility; and 5, moribund. Blood obtained from the submandibular vein was used for virus titration. Periodic blood samples were collected from the tail vein via a prick for glucose and lactate measurements using Accu-Chek and LACTATE PRO2 LT-1730 devices, respectively.

### Streptozotocin-induced diabetic mice

Diabetic mellitus in mice was induced using a single high dose of streptozotocin, following a previously described protocol (12). Briefly, female *Stat1^-/-^* mice were fasted for 4 h prior to receiving an intraperitoneal injection of streptozotocin (200 mg/kg in sodium citrate buffer, pH 4.5). After injection, the mice were given regular food and 10% sucrose water for 12 h, followed by regular water the next day.

### Antibodies, chemicals, and reagents

The MitoProbe Jc-1 Assay Kit (Invitrogen, USA) was used to detect mitochondrial depolarization, indicated by an increase in green fluorescence (Jc-1 monomer), following the manufacturer’s instructions. Mag-Fluo-4 AM (Invitrogen) and Rhod-2 AM (Invitrogen) were used to stain calcium in the ER and mitochondria, respectively. Antibodies for AIF (ab32516, Abcam, UK), PDI (#3501, Cell Signaling Technology, USA), and DENV E (YH0026, Yao-Hong Biotechnology, Taiwan) were used for immunostaining, and DAPI (H-1200, VECTASHIELD, USA) was used to label nuclear DNA. Cell images were captured using an Olympus IX73 microscope or a Leica TCS SP5II confocal microscope at the NHRI Core Instrument Center. Antibodies for p-eIF2a (CST3398, Cell Signaling Technology), LC3B (GTX127375, GeneTex, Taiwan), GRP78 (CST3177, Cell Signaling Technology), HSP70 (GTX111088, GeneTex), Erp44 (CST3798, Cell Signaling Technology), Actin (GTX109639, GeneTex), DENV E (GTX127277, GeneTex), p-PKR (sc-101783, Santa Cruz, USA), and PKR (sc-707, Santa Cruz) were used for immunoblotting. Salubrinal (S2923, Selleckchem) was used to inhibit eIF2α phosphatase. Oxamate (O2751, Sigma) was used to block lactate dehydrogenase activity.

### Transmission electron microscope (TEM)

DENV-infected cells were fixed with 2.5% glutaraldehyde for 30 min and processed at the Electron Microscope Division, Cell Biology Core Lab, Institute of Plant and Microbial Biology, Academia Sinica. Cells were examined using a Transmission Electron Microscope (FEI Tecnai G2 Spirit, 2014). For quantification, approximately 50 images per sample grid were captured using a Borries Optimus 100 Scanning TEM (STEM) at an accelerating voltage of 30 kV. Each image had a field of view of approximately 80 µm × 80 µm. Images were processed using Fiji software with histogram equalization and gamma adjustment to enhance image quality. A total of 500–600 cells were analyzed, and the percentages of cells containing small vacuoles, large vacuoles, or exhibiting ER aggregation were recorded.

### Quantitative real-time (RT)-PCR

The detailed information for RNA extraction, cDNA synthesis, and quantitative real-time PCR has been described previously (4). The primer sequences used in the study were: β-Actin(*Actb*): Forward gctacagcttcaccaccaca, Reverse aaggaaggctggaaaagagc; Glucose transporter protein type 1(*Glut1*): Forward cttgcttgtagagtgacgatc, Reverse cagtgatccgagcactgctc; Hexokinase 2 (*Hk2*) : Forward attcctgcctgatactgcc, Reverse acacacacacacacacatac; Phosphoglycerate kinase 1 (*Pgk1*): Forward gctgttccaagcatcaaattc, Reverse tcttttcccttcccttcttcc; Lactate dehydrogenase A (*Ldha*): Forward ggcactgacgcagacaag, Reverse tgatcacctcgtaggcactg; Relative mRNA expression levels were calculated using the ΔΔCq method with *Actb* cDNA as an internal control.

### Statistical analysis

Unless otherwise indicated, data are presented as mean ± SD. Statistical differences between treatment groups were analyzed using the Mann–Whitney *U* test, one-way ANOVA, two-way ANOVA, unpaired *t*-test, or the log-rank (Mantel–Cox) test, as appropriate for each experimental design. *P < 0.05, **P < 0.01.

## Data Availability

The data that support the findings of this study are available from the corresponding author upon reasonable request.

## SUPPLEMENTAL MATERIAL

Supplemental figures jvi.01177-25-s0001.docx Figures S1 to S10.

ASM does not own the copyrights to Supplemental Material that may be linked to, or accessed through, an article. The authors have granted ASM a non-exclusive, world-wide license to publish the Supplemental Material files. Please contact the corresponding author directly for reuse.
