# Supplementary material for: Pathogenic dengue virus TW2015 strain infection triggers anaerobic glycolysis and enhances mortality in diabetic mice
Source: J Virol. 2025 Nov 10;99(12):e01177-25. doi: 10.1128/jvi.01177-25 (PMC12724219; doi:10.1128/jvi.01177-25)
Supplement: Supplemental figures — Figures S1 to S10. [file jvi.01177-25-s0001.docx]

**Supplementary Figures**

**
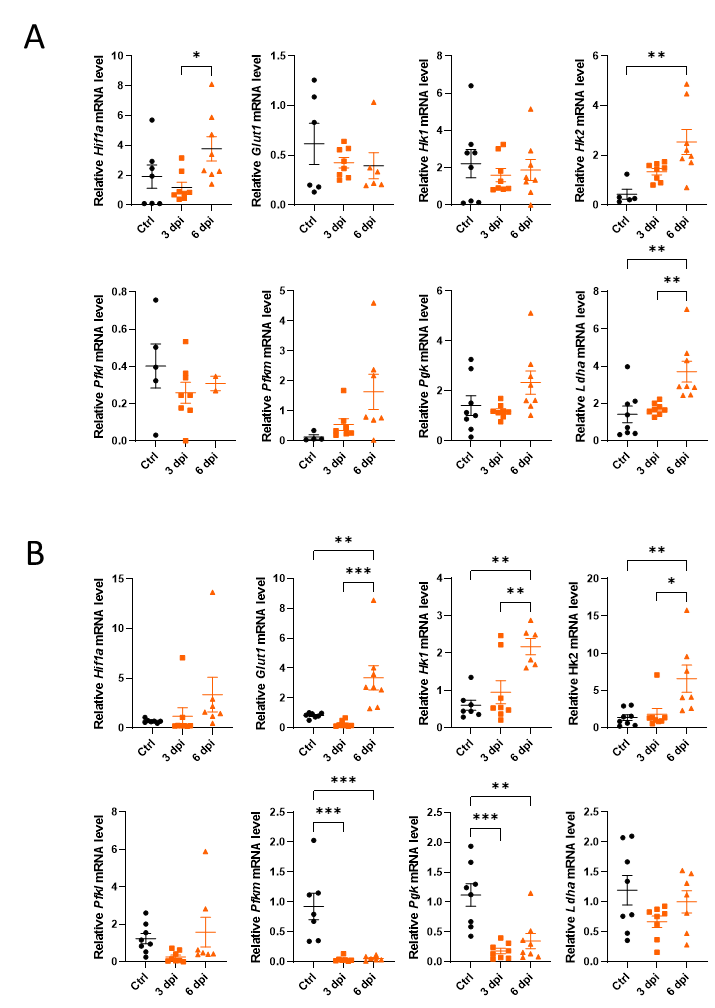
**

**Fig. S1** *Stat1^-/-^* mice were infected intravenously with DENV-2 TW2015 (n=8) strains (5×10^4^ PFU/mouse). Mouse organs were collected on 3 and 6 dpi (days-post infection). Mouse organs were collected at 3 and 6 days post-infection (dpi). Spleens (A) and livers (B) were analyzed by RT-qPCR to assess the expression of glycolysis-related genes. Statistical analyses: ordinary one-way ANOVA. p < 0.05 (*), p < 0.01 (**), p < 0.001 (***). Hypoxia-inducible factor 1-α (*Hif1a*); Glucose transporter protein type 1(*Glut1*); Hexokinase 1 (*Hk1*); Hexokinase 2 (*Hk2*); Phosphoglycerate kinase (*Pgk*); Muscle subunit of the enzyme phosphofructokinase (*Pfkm*); Liver type of PFK (*Pfkl*); Lactate dehydrogenase A (*Ldha*).


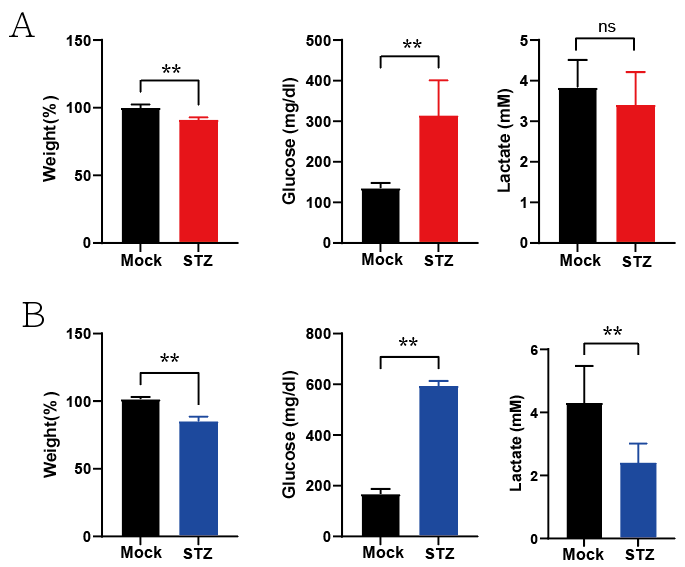


**Fig. S2** Streptozotocin-indecd diabetic mice. (A) Female *Stat1^-/-^* mice were treated with streptozotocin (STZ, 200 mg/kg, i.p., n=6). Body weight, blood glucose, and lactate levels were monitored for 7 days, with data shown from Day 7. (B) Male *Stat1^-/-^* mice (n = 9) were treated with STZ under the same conditions. Mann-Whitney tests. ns: no significance. p < 0.01 (**).


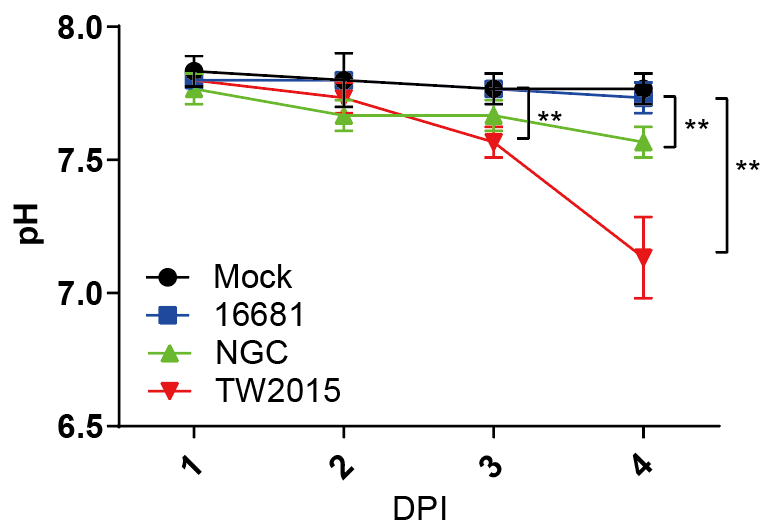


**Fig. S3** Vero cells were infected with DENV-2 strains (16681, NGC and TW2015; m.o.i.=0.1) and the culture medium pH was measured using a a Compact pH meter (LAQUAtwin-pH-11). Statistical analysis was performed using two-way ANOVA with Tukey’s multiple comparisons test; ** p < 0.01.

**
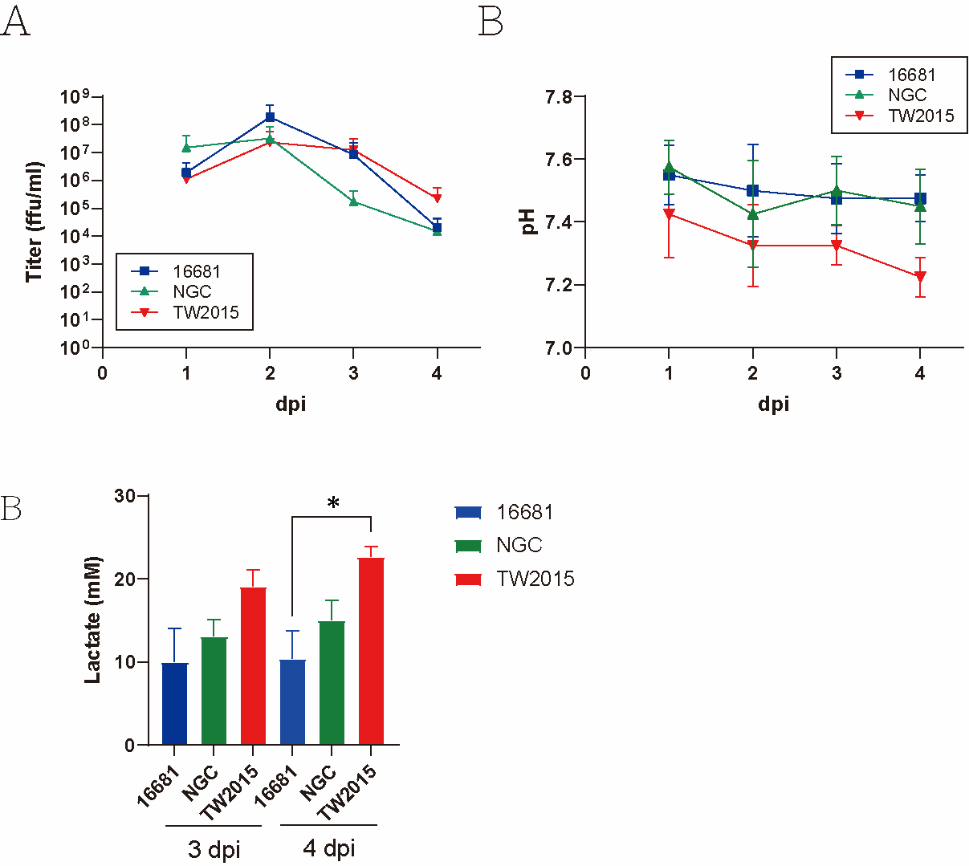
**

**Fig. S4** Huh7 cells were infected with DENV-2 strains 16681, NGC, or TW2015 (n=3). The virus titer (A), pH (B) and lactate level (C) in the culture medium were monitored. The data were collected from three independent experiments. Statistical analysis was performed using one-way ANOVA. * p < 0.05.

**
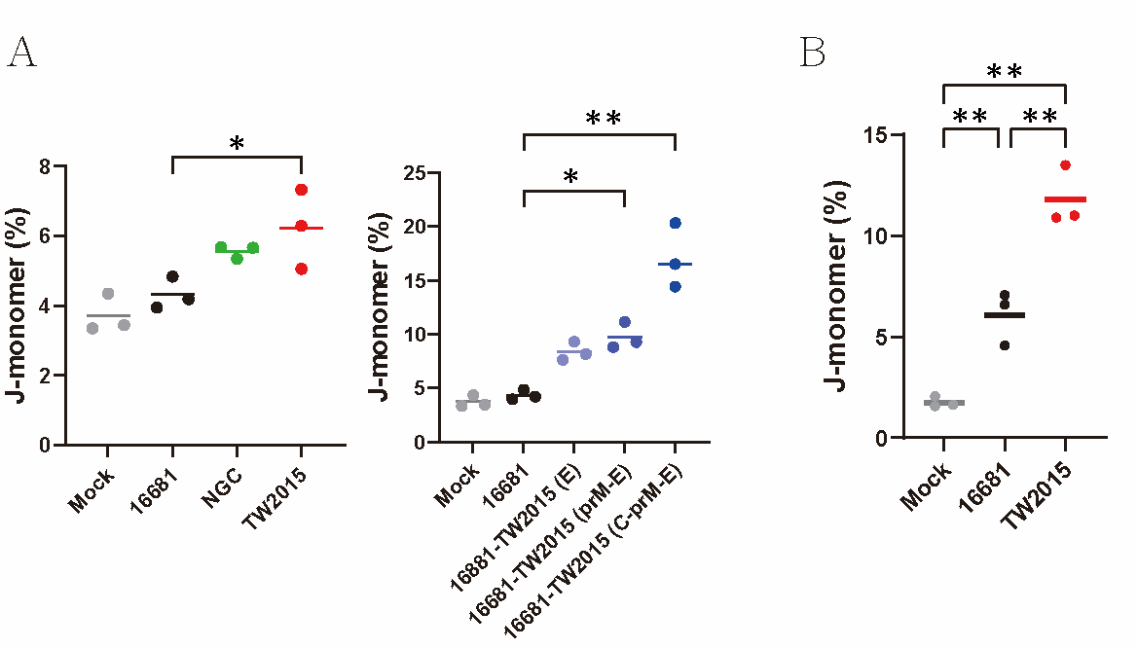
**

**Fig. S5** (A) Vero cells were infected with DENV-2 viruses or 16681-based recombinant viruses carrying TW2015 segments. The infected cells were stained with JC-1 dye at 2 dpi, and the percentage of cells with dysfunctional mitochondria (J-monomer+) was measured by flow cytometry (n=3). (B) Huh7 cells infected with DENV-2 viruses were stained with JC-1 dye at 3 dpi for flow cytometry analysis (n=3). Statistical significance was determined using one-way ANOVA; * *p* < 0.05, ** *p* < 0.01.

**
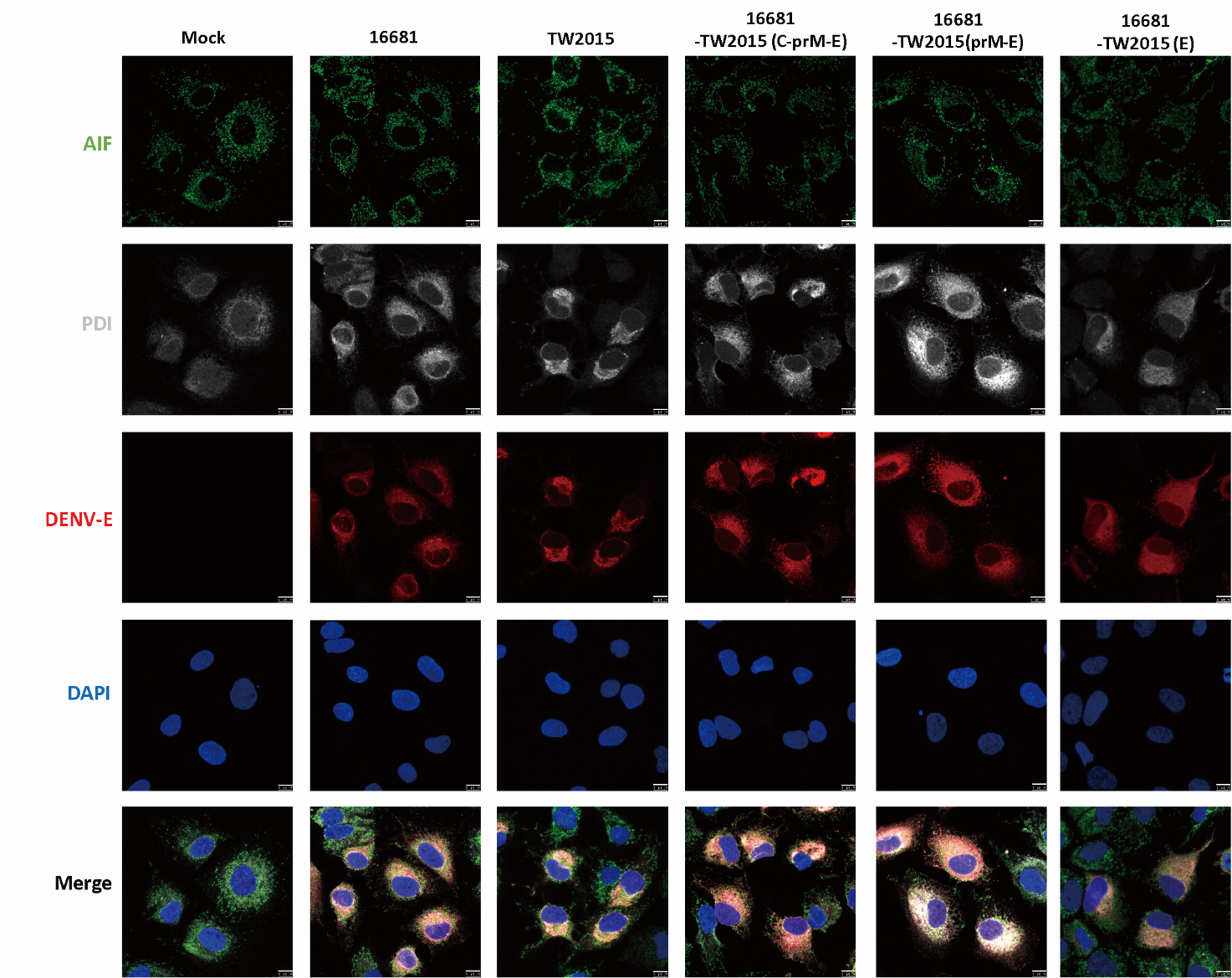
**

**Fig. S6** Huh7 cells were infected with DENV-2 strains 16681, TW2015, or recombinant viruses for 48 hours. The cells were subjected to immunostaining using specific antibodies against markers for ER (PDI), mitochondria (AIF), and the DENV envelope. Nuclear DNA was counterstained with DAPI dye, and images were captured using a confocal microscope. Scale bar:10μm.

.

(A)


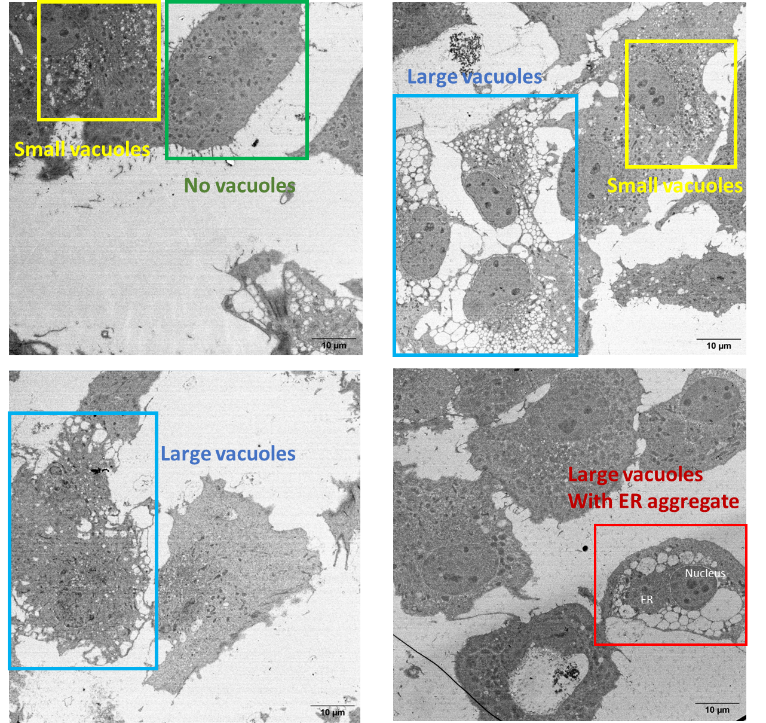


(B)


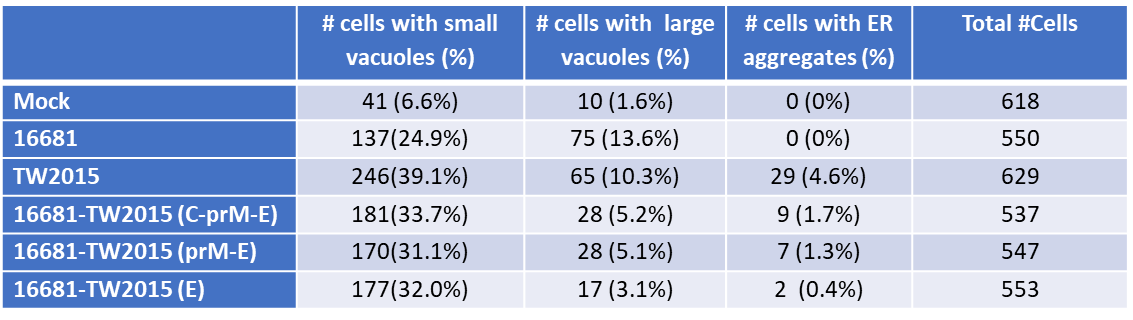


**Fig. S7 (A)** Representative electron microscopy images of DENV-infected cells displaying vacuolar and ER morphological changes. Electron microscopy images were acquired using a Borries K004 STEM at an accelerating voltage of 30 kV. Cells were classified based on vacuole presence and ER morphology: green box indicates cells with no visible vacuoles; yellow box, cells with small vacuoles; blue box, cells with large vacuoles; and red box, cells exhibiting condensed ER aggregates. Scale bar: 10 μm. (B) Quantification results.

**
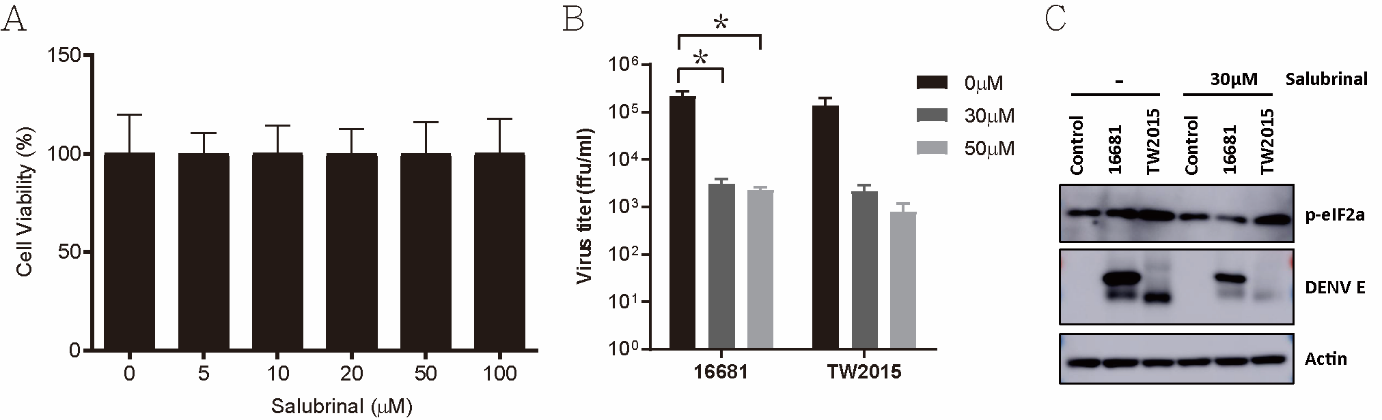
**

**Fig. S8** (A) Huh7 cells were treated with Salubrinal, an eIF2α phosphatase inhibitor, for 48 hours, and cell viability was measured by MTT assay (n=4). (B) Huh7 cells were infected with DENV-2 strains 16681 or TW2015 in the presence of Salubrinal (30 or 50 μM; n=3). The virus titer at 2 dpi was measured. (C) DENV-2-infected cells (2 dpi) treated with Salubrinal (30 μM) were subjected to immunoblotting. Statistical significance was determined using one-way ANOVA; * *p* < 0.05.

**
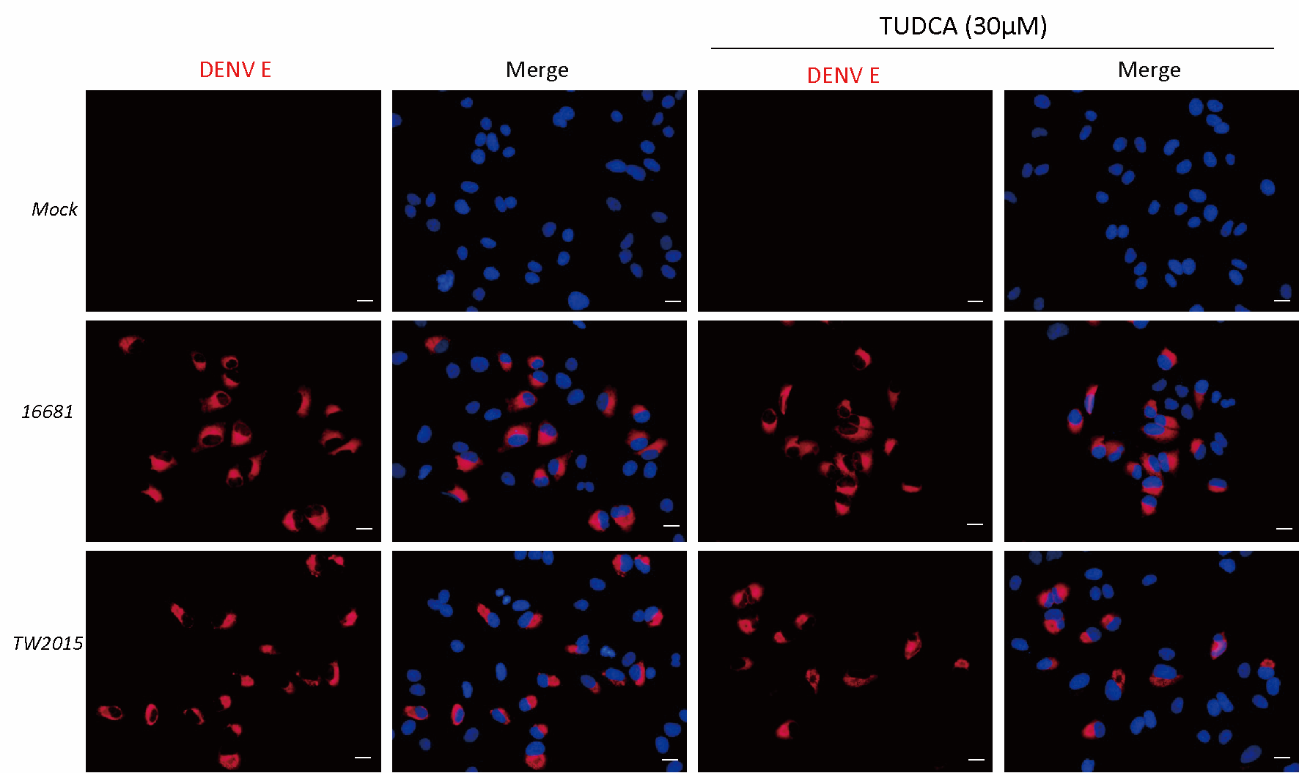
**

**Fig. S9** Huh7 cells were infected with DENV-2 strains 16681 or TW2015 viruses for 24 hours and further incubated with TUDCA (30 μM, Selleckchem, USA), a chemical chaperone, for another 24 hours. The cells were then fixed and stained with an anti-E antibody. Scale bar: 20μm.

**
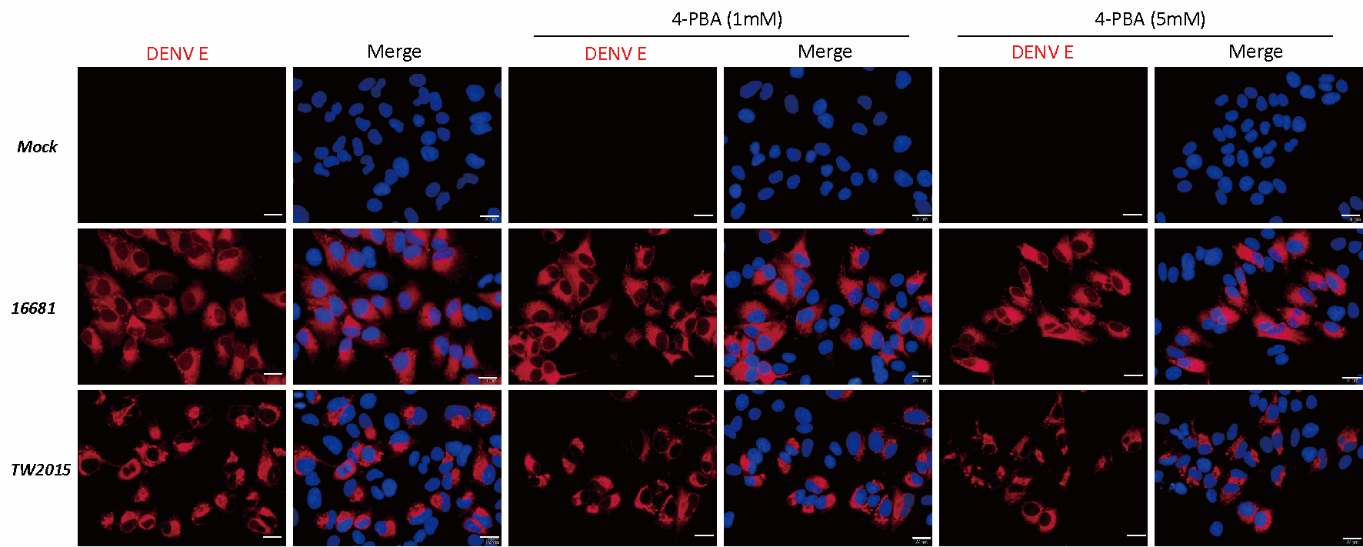
**

**Fig. S10** Huh7 cells were infected with DENV-2 strains 16681 or TW2015 viruses for 24 hours and further incubated with 4-PBA (1 or 5 mM; Sigma), a chemical chaperone, for another 24 hours. The cells were then fixed and stained with an anti-E antibody. Scale bar: 20μm.
